# Virological outcomes, resistance profile and viral evolution of lenacapavir-based regimens in heavily treatment-experienced people: data from the PRESTIGIO Registry

**DOI:** 10.1093/jac/dkag335

**Published:** 2026-10-28

**Authors:** Maria Mercedes Santoro, Daniele Spalletta, Vincenzo Spagnuolo, Chiara Paletti, Greta Marchegiani, Maria Concetta Bellocchi, Riccardo Lolatto, Luca Carioti, Adriana Cervo, Antonio Di Biagio, Marta Fiscon, Emanuele Focà, Roberta Gagliardini, Stefano Rusconi, Maurizio Zazzi, Antonella Castagna, Francesco Saladini, Francesca Ceccherini-Silberstein, PRESTIGIO Registry, Antonella Castagna, Vincenzo Spagnuolo, Daniele Armenia, Stefano Bonora, Leonardo Calza, Anna Maria Cattelan, Giovanni Cenderello, Adriana Cervo, Laura Comi, Antonio Di Biagio, Emanuele Focà, Roberta Gagliardini, Andrea Giacomelli, Filippo Lagi, Giulia Marchetti, Stefano Rusconi, Francesco Saladini, Maria Santoro, Maurizio Zazzi, Andrea Galli, Daniele Armenia, Francesco Saladini, Maria Santoro, Maurizio Zazzi, BioRep SRL, Elisabetta Carini, Sabrina Bagaglio, Girolamo Piromalli, Riccardo Lolatto, Nicolò Capra, Marcello Riccardo Tavio, Alessandra Mataloni Paggi, Silvia Magnani, Manuela Colafigli, Ornella Schioppa, Stefania Zanussi, Valentina Da Ros, Silvia Rossetto, Annalisa Saracino, Flavia Balena, Laura Comi, Daniela Valenti, Pierluigi Viale, Leonardo Calza, Maddalena Giglia, Claudio Rigamonti, Silvia Cretella, Francesco Castelli, Emanuele Focà, Davide Minisci, Stefano Rapino, Barbara Menzaghi, Maddalena Farinazzo, Chiara Abeli, Bruno Cacopardo, Maurizio Celesia, Michele Salvatore Paternò Raddusa, Carmen Giarratana, Paolo Fusco, Vincenzo Olivadese, Simona Mongiardi, Angelo Pan, Chiara Fornabaio, Paola Brambilla, Alessandro Bartoloni, Filippo Lagi, Paola Corsi, Sasha Trevisan, Giuseppe Gasparro, Cecilia Costa, Alessio Bellucci, Elisa Mariabelli, Teresa Santantonio, Sergio Lo Caputo, Sergio Ferrara, Arianna Narducci, Emanuele Pontali, Marcello Feasi, Antonio Sarà, Matteo Bassetti, Antonio Di Biagio, Sabrina Blanchi, Stefania Piconi, Martina Bottanelli, Silvia Pontiggia, Giada Valsecchi, Stefano Rusconi, Cinzia Roberta Bassoli, Francesco Bassani, Liana Bevilacqua, Antonella Castagna, Vincenzo Spagnuolo, Riccardo Lolatto, Nicolò Capra, Rebecka Papaioannu, Tommaso Clemente, Elisabetta Carini, Girolamo Piromalli, Sabrina Bagaglio, Andrea Galli, Camilla Muccini, Golnaz Torkjazi, Spinello Antinori, Andrea Giacomelli, Tiziana Formenti, Giacomo Pozza, Giulia Marchetti, Lidia Gazzola, Fabiana Trionfo Fineo, Massimo Puoti, Cristina Moioli, Federico D’Amico, Simoncini Elena, Sassi Serena, Cristina Mussini, Adriana Cervo, Giulia Nardini, Elio Manzillo, Antonella Gallicchio, Anna Maria Cattelan, Antonio Cascio, Marcello Trizzino, Elisa Fronti, Diletta Laccabue, Federica Carli, Roberto Gulminetti, Layla Pagnucco, Mattia Demitri, Alessandra Ferrari, Daniela Francisci, Giuseppe De Socio, Elisabetta Schiaroli, Elisa Garlassi, Romina Corsini, Roberta Gagliardini, Marisa Fusto, Loredana Sarmati, Vincenzo Malagnino, Tiziana Mulas, Mirko Compagno, Carlo Torti, Simona Di Giambenedetto, Silvia Lamonica, Pierluigi Salvo, Giovanni Cenderello, Rachele Pincino, Davide Laurenda, Giordano Madeddu, Andrea De Vito, Mario Tumbarello, Massimiliano Fabbiani, Francesca Panza, Ilaria Rancan, Giovanni Di Perri, Stefano Bonora, Micol Ferrara, Andrea Calcagno, Silvia Fantino, Giancarlo Orofino, Guido Calleri, Marta Guastavigna, Stefano Nardi, Marta Fiscon

**Affiliations:** Department of Experimental Medicine, University of Rome ‘Tor Vergata’, Via Montpellier 1, Rome 00133, Italy; Department of Experimental Medicine, University of Rome ‘Tor Vergata’, Via Montpellier 1, Rome 00133, Italy; Infectious Diseases, IRCCS San Raffaele Scientific Institute, Milan, Italy; Department of Medical Biotechnologies, University of Siena, Siena, Italy; Department of Experimental Medicine, University of Rome ‘Tor Vergata’, Via Montpellier 1, Rome 00133, Italy; Department of Experimental Medicine, University of Rome ‘Tor Vergata’, Via Montpellier 1, Rome 00133, Italy; Infectious Diseases, IRCCS San Raffaele Scientific Institute, Milan, Italy; Department of Experimental Medicine, University of Rome ‘Tor Vergata’, Via Montpellier 1, Rome 00133, Italy; Infectious Disease Clinic, University of Modena and Reggio Emilia, AOU Policlinico di Modena, Modena, Italy; Infectious Diseases Unit, San Martino Hospital Genoa, University of Genoa, Genoa, Italy; Infectious Diseases Unit, Azienda Scaligera di Verona, Verona, Italy; Department of Clinical and Experimental Sciences, University of Brescia-ASST Spedali Civili, Brescia, Italy; Clinical and Research Infectious Diseases Department, National Institute for Infectious Diseases Lazzaro Spallanzani IRCCS, Rome, Italy; S.C. Malattie Infettive, ASST Ovest Milanese, Legnano General Hospital, Legnano, Italy; Department of Biomedical and Clinical Sciences, Università Degli Studi di Milano, Milan, Italy; Department of Medical Biotechnologies, University of Siena, Siena, Italy; Infectious Diseases, IRCCS San Raffaele Scientific Institute, Milan, Italy; Infectious Diseases Unit, Vita-Salute San Raffaele University, Milan, Italy; Department of Medical Biotechnologies, University of Siena, Siena, Italy; Department of Experimental Medicine, University of Rome ‘Tor Vergata’, Via Montpellier 1, Rome 00133, Italy

## Abstract

**Background:**

Lenacapavir (LEN) is a first-in-class, long-acting HIV-1 capsid inhibitor, approved for the treatment of heavily treatment-experienced (HTE) people with HIV (PWH) with multidrug resistance (MDR). To date, few data are available on resistance profiles in real life.

**Methods:**

Paired plasma and PBMC samples from nine HTE PWH with MDR enrolled in the PRESTIGIO Registry receiving LEN in combination with an optimized background regimen were collected before (baseline) and during LEN treatment. Next-generation sequencing was performed using in-house protocols. LEN susceptibility was assessed by single-cycle assay. Intra-patient viral evolution was evaluated through quasispecies distance analysis.

**Results:**

At baseline, no major LEN resistance-associated mutations were detected; the accessory capsid polymorphism T107A was identified as a minority variant (5.9%) in one case, with phenotypic assays confirming full LEN susceptibility (mean fold-change: 1.2). Participants received LEN for a median [IQR] of 19.8 [18.5–23.2] months. Two individuals started LEN with undetectable viremia and maintained suppression. The other seven participants were viremic; six achieved viral suppression, with one experiencing a blip. One low-adherent participant exhibited persistent viremia with Q67K and K70H as the majority variants in both plasma (98.5%; 97.8%) and PBMCs (75.7%; 75.6%), conferring a 2081-fold reduction in LEN susceptibility. Intra-host variability in the capsid and *pol* regions remained low over time and across compartments.

**Conclusions:**

In this case series of HTE PWH with MDR, LEN was highly associated with maintenance or achievement of virological suppression and limited virus evolution, with only one participant remaining viremic and showing a majority highly LEN-resistant variant.

## Background

People with HIV-1 who are heavily treatment-experienced (HTE) and harbour a multidrug-resistant (MDR) virus face limited therapeutic options and a high risk of virological failure. Therefore, innovative strategies are needed to suppress viral replication and preserve immune function in this fragile population. In this context, the new first-in-class, long-acting capsid inhibitor lenacapavir (LEN) has been recently approved for the treatment of HTE people with MDR, in combination with an optimized background regimen (OBR).^1,2^ This drug demonstrated potent antiviral activity by binding to the HIV capsid protein and interfering with early and late viral replication steps, including the nuclear import of proviral DNA and the coordinated assembly and maturation of the capsid core.^3–6^ A significant benefit of LEN therapy is its long-acting formulation, which allows subcutaneous administration every six months after an oral loading phase, addressing adherence problems. In addition to treatment, LEN has also demonstrated efficacy in prevention,^7,8^ and, for this reason, has been recently approved by the FDA for its use in pre-exposure prophylaxis (PrEP). Despite its many advantages, several resistance-associated mutations (RAMs) in the capsid region have been selected by this drug, both *in vitro* and *in vivo*. Notably, *in vitro* studies have reported the emergence of specific mutations, including Q67H, N74D, M66I, K70N and T107N, under LEN selective pressure.^5,9^ Data from the CAPELLA clinical trial have also reported cases of virological failure associated with these capsid mutations in HTE individuals with MDR HIV, further underscoring the clinical relevance of these mutations.^10^

Notably, the N74D mutation has been reported to emerge rapidly *in vivo*, as early as week 3 of treatment, especially when LEN is administered without fully active background therapy.^11^ Similarly, in the PURPOSE 2 trial evaluating LEN for PrEP, N74D emerged in both participants who acquired HIV despite adequate drug exposure, suggesting that monotherapy with LEN during early infection can rapidly select this resistance mutation.^8^ All these above-mentioned mutations can lead to a reduced LEN susceptibility,^9^ emphasizing the need for OBR and sustained adherence. Given these concerns, evaluating resistance in the capsid could be useful for optimizing LEN-based regimens, particularly in HTE populations with advanced resistance profiles and limited options. In this context, real-world data are crucial to inform clinical decisions.

The aim of this study was to evaluate the genotypic and phenotypic resistance profile in HTE PWH with MDR enrolled in the Italian PRESTIGIO Registry and treated with LEN. The intra-patient viral evolution over time and between compartments (plasma and peripheral blood mononuclear cells [PBMCs]) was also explored, with a focus on genetic variability within the capsid, protease/reverse transcriptase and integrase regions.

## Materials and methods

### Participants

Participants included in the present study were selected from the PRESTIGIO Registry (https://registroprestigio.org/; ClinicalTrials.gov ID NCT04098315), an Italian registry that collects clinical and laboratory data of people with HIV (PWH) with resistance to the main four classes of antiretroviral drugs: nucleos(t)ide reverse transcriptase inhibitors (NRTIs), non-nucleoside reverse transcriptase inhibitors (NNRTIs), protease inhibitors (PIs) and integrase strand transfer inhibitors (INSTIs).^12^ During the study period, 253 individuals were enrolled in the PRESTIGIO Registry, of whom 205 were actively followed up. All the study participants were starting a LEN-based regimen for the first time; for all of them, paired plasma and PBMC samples were collected at baseline (T0, defined as the last available sample before LEN initiation), when available, and at follow-up (T1, after a minimum of six months of continuous LEN treatment). The flow of samples analysed and the experiments performed are described in Figure 1 and in Table S2 (available as Supplementary data at *JAC* Online).

**Figure 1.**
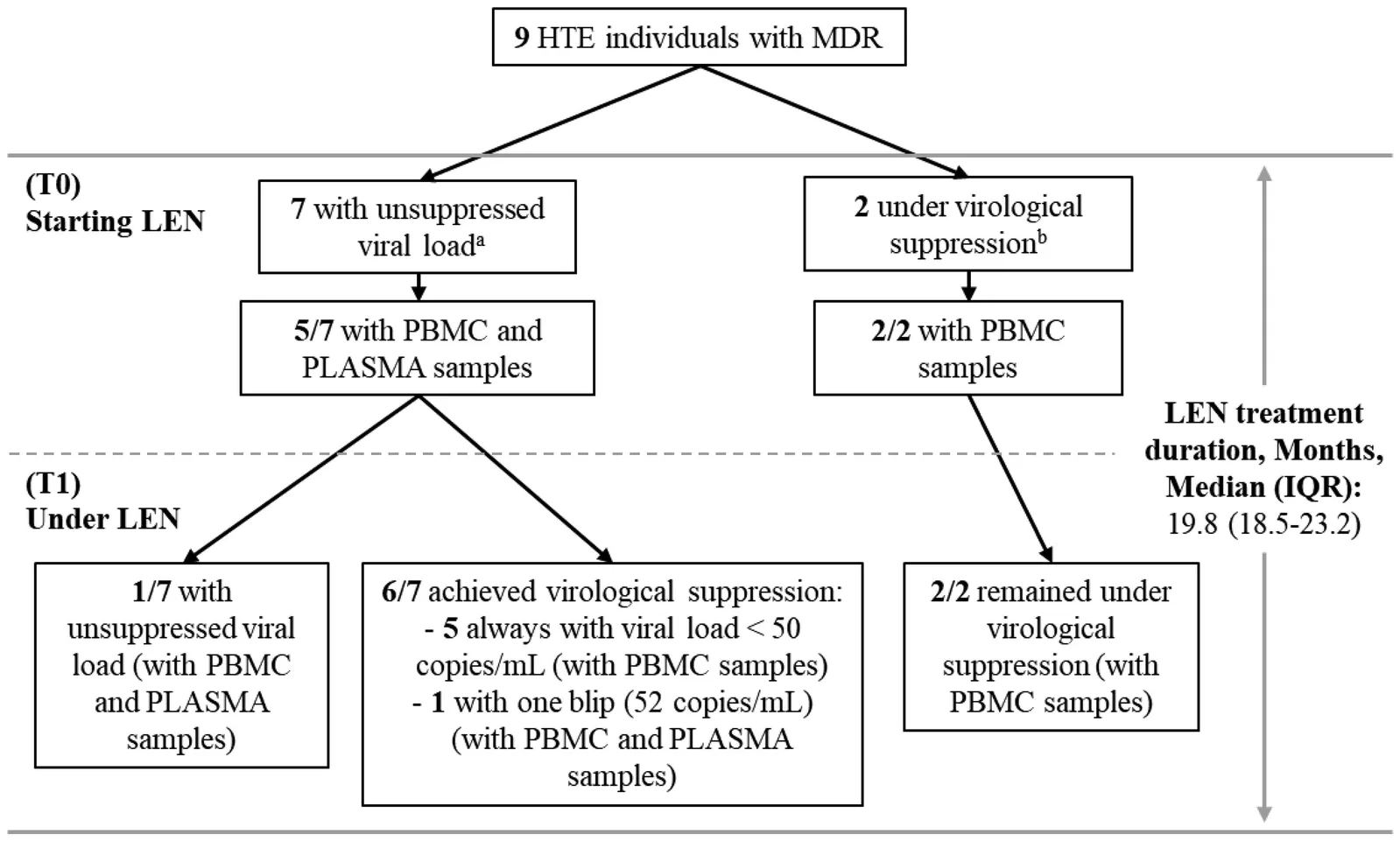
Flow-chart of the nine HTE-MDR individuals who started LEN for whom genotypic and phenotypic resistance profiles were evaluated. ^a^Plasma HIV-1 RNA >50 copies/mL. ^b^Plasma HIV-1 RNA <50 copies/mL. Samples available at baseline were collected in a period ranging from 90 days before to 7 days after LEN start.

### Ethics

The PRESTIGIO Registry was approved by the Ethics Committee of the coordinating centre (IRCCS San Raffaele Scientific Institute, Milan, Italy, protocol number 41/int/December 2017) and by the Ethics Committees of all the participating centres. All patients provided written informed consent to be enrolled.

### NGS genotyping on plasma and PBMC samples

Viral RNA was extracted using the QIAamp Viral RNA Mini Kit (QIAGEN, Hilden, Germany) following the manufacturer’s protocol. Total DNA was extracted from PBMCs using a commercially available kit (High Pure PCR Template Preparation Kit, Roche) following the manufacturer's instructions. Next-generation sequencing (NGS) was conducted using in-house protocols for the target regions, including the capsid (CA)^13^ and *pol* gene (protease, PR; reverse transcriptase, RT; integrase, IN), as previously described.^14^ DNA libraries were prepared with the Nextera XT DNA Library Prep Kit and sequenced on the MiSeq platform (Illumina Inc.).

### NGS data analyses, evaluation of resistance and subtyping

After sequencing, NGS data with a minimum coverage of 500 reads per position were analysed using an NGS variant detection threshold set at 5%. Specifically, we adopted a minimum coverage requirement of 500 reads per position to ensure robust and reliable detection of low-frequency variants. A 5% threshold for NGS mutation detection was adopted based on previous evidence indicating that this cut-off ensures good agreement with conventional genotyping while limiting the influence of low-frequency sequencing artefacts.^13,15,16^ The Stanford HIVdb algorithm (HIVdb version 9.8, https://hivdb.stanford.edu) was used to evaluate the resistance to LEN, PIs, RTIs and INSTIs and to calculate the genotypic susceptibility score (GSS) of the start OBR administered together with LEN. The overall GSS of the OBR was calculated as the sum of the GSSs for all individual drugs included in the regimen by considering all the genotypes available before LEN start, including the genotype performed by NGS at baseline; details on the GSS calculation are provided as a note in Table 1. HIV-1 subtyping was determined on the *pol* gene by using both the automatic tool COMET (https://comet.lih.lu/) and phylogenetic analysis. Specifically, a maximum likelihood phylogenetic tree was constructed using IQ-TREE2 software (v2.1.3) with 1000 bootstrap replicates.

**Table 1.** Participants’ characteristics at baseline (T0)

Characteristics at baseline^a^	Overall (*n* = 9)
Age, years, Median (IQR)	55 (45–65)
Male, n (%)	8 (88.9)
Zenith Viremia, Log_10_ copies/mL, Median (IQR)^b^	5.5 (5.1–5.7)
Nadir CD4+ T-cells, cells/mm^3^, Median (IQR)	52 (28–137)
CD4+ T-cells, cells/mm^3^, Median (IQR)	187 (148–469)
Plasma HIV-1 RNA <50 copies/mL, n (%)	2 (22.2)
Plasma HIV-1 RNA >50 copies/mL, n (%)	7 (77.8)
Plasma HIV-1 RNA, copies/mL, Median (IQR)^c^	2010 (763–24 400)
X4 tropism, n (%)^c^	4 (50.0)
HIV-1 subtype, n (%)	
B	8 (88.9)
CRF05_DF	1 (11.1)
Duration of HIV infection, years, Median (IQR)	29 (29–31)
Duration of ART, years, Median (IQR)	27 (23–29)
Number of previous regimens, Median (IQR)	28 (26–41)
Previous drug class exposure, n (%)	
NRTI	9 (100)
NNRTI	8 (88.9)
PI	9 (100)
INSTI	9 (100)
FI^d^	8 (88.9)
EI^e^	8 (88.9)
Number of drugs previously administered per class, median (IQR)	
NRTI	6 (4.5-7)
NNRTI	2 (1.5–3.5)
PI	6 (3–6)
INSTI	2 (2–2.5)
EI^e^	1 (1–2.5)
Calendar year of LEN start, Median (IQR)	2020 (2020–2021)
Number of drugs administered with LEN, n (%):	
*3*	1 (11.1)
*>3*	8 (88.9)
GSS of the background regimen	
*Median (IQR)*	1.5 (1.0–2.5)
*Min-Max*	0.0–4.0

^a^Baseline: Before lenacapavir (LEN) start.

^b^Data available for eight participants.

^c^Median and IQR were calculated on the seven participants that had plasma HIV-1 RNA >50 copies/mL.

^d^Enfuvirtide.

^e^Fostemsavir administered to four participants; ibalizumab administered to three participants; maraviroc administered to seven participants. GSS, genotypic susceptibility score, calculated on all the antiretrovirals in the regimen with LEN, by considering all the genotypes available before LEN start, including the genotype performed by NGS. GSS for single drugs was scored as 0 (High-level resistance), 0.5 (Intermediate resistance and Low-level resistance) and 1 (Potential low-level resistance and Susceptible), according to the Stanford rules (https://hivdb.stanford.edu/page/release-notes/#algorithms). In the case of maraviroc-containing regimens, the drug was only considered active (GSS = 1) in individuals with a CCR5 virus. For regimens including enfuvirtide, fostemsavir or ibalizumab, these drugs were considered active (GSS = 1) only if never previously administered or unless prior treatment failure was documented, and partially active (GSS = 0.5) in case of previous documented treatment failure.

### Evaluation of viral evolution under LEN pressure

To investigate the differential evolution dynamics of viral quasispecies across biological compartments and over time, the distances between viral populations derived from plasma (HIV-1 RNA) and PBMCs (proviral HIV-1 DNA) were calculated using the Quasispecies Distance tool available in Galaxy (www.usegalaxy.fr).^17^ This tool estimates pairwise distances between multiple quasispecies samples by analysing read pileups aligned to the same genomic region. It computes the cosine relatedness between viral populations, reporting angular cosine distance or cosine similarity as index for evolutionary divergence. These metrics provide an approximation of the genetic distance between quasispecies, reflecting their degree of relatedness. Quasispecies distance (Q-distance) was estimated separately for the PR/RT, IN and CA regions.

### Evaluation of *in vitro* susceptibility to LEN

*In vitro* susceptibility to LEN was measured through a single-cycle assay in TZM-bl cells using NL4-3-based recombinant viruses. For the amplification of the *gag*-PR coding region (HXB2 nucleotide coordinates 522-2764), reverse transcription and the first round of PCR from 10 µL of extracted RNA were performed using the Superscript III One-Step RT-PCR system (Thermo Fisher Scientific), and then nested PCR was performed using the Q5 High-Fidelity DNA Polymerase (NEB) according to the manufacturer’s instructions. The generation of recombinant viruses harbouring a clinically derived *gag*-PR coding region was previously described.^18^ Briefly, triplicate nested PCR was co-transfected with linearized pNL4-3ΔGAG-PR in Lenti-X 293T cells using the calcium-phosphate approach. Supernatants harbouring recombinant viruses were harvested 48 hours post-transfection and expanded in MT-2 cells to increase viral titers. In the presence of large cellular syncytia, supernatants were harvested and stored at −80°C. To determine the half-maximal inhibitory concentration (IC_50_), TZM-bl cells were seeded in a 96-well plate at a concentration of 15 000 cells per well and infected with 750 TCID_50_ per well of virus in the presence of 5-fold serial dilutions of LEN. After 48 hours, cells were lysed adding 40 µL/well of Glo-Lysis Buffer (Promega), and the lysate was transferred to a luminescence plate. Forty microlitres of Bright-Glo Luciferase Reagent (Promega) were added to each well, and luminescence was measured in the Glomax Discover platform (Promega). Relative Luminescence Units (RLU) detected in each well were elaborated with GraphPad Prism version 5.0 (GraphPad Software) to calculate the IC_50_ values, then fold-change (FC) values were calculated as the ratio between the IC_50_ of each recombinant virus and the IC_50_ of the NL4-3 wild-type virus.

### Statistical analysis

Descriptive statistics were used to analyse participants’ characteristics at baseline (T0). Regarding the evaluation of genetic evolution from T0 to T1, the comparison of Q-distances across timepoints was performed using non-parametric tests due to the non-normal distribution of the data. Specifically, the Kruskal–Wallis test was used to evaluate global differences among the different regions, while pairwise comparisons between gene regions were assessed using the Dunn’s test with Bonferroni correction to adjust for multiple comparisons. A two-sided *P*-value ≤0.05 was considered statistically significant.

## Results

### Study population

Among the nine individuals that were enrolled in the study, seven started LEN with plasma HIV-1 RNA >50 copies/mL (median [IQR] 2010 [763–24 400] copies/mL), while two were with plasma HIV-1 RNA <50 copies/mL (Figure 1, Table 1). Eight participants harboured HIV-1 subtype B and one harboured the CRF05_DF recombinant variant. Participants had a long treatment history, with a median ART duration of 27 years (IQR, 23–29), almost all of them had prior exposure to all drug classes used in clinical practice (median [IQR] number of drug classes: 6 [5.5–6]) (Table 1).

The median GSS of the OBR administered with LEN at baseline was 1.5 [IQR, 1.0–2.5], with a Min-Max range of 0.0–4.0. Specifically, five out of nine participants (55.6%) initiated LEN with a GSS <2.0, with one of them (ID-03) presenting a GSS of 0.0. Eight participants (88.9%) received a regimen including LEN together with more than three antiretroviral agents. The median duration of LEN treatment was 19.8 (IQR, 18.5–23.2) months. During this period, participants changed their OBR in a median number of 1 (IQR, 0–1). Specifically, of the nine participants, three did not undergo any change, one change occurred in four participants, two changes occurred in one and four changes in another. Detailed individual drug scoring, the exact number of active compounds, and details of the drugs co-administered with LEN are reported in Table S1.

Among the seven participants who started LEN with detectable viremia, six achieved virological suppression, with one showing a viral blip (52 copies/mL). The seventh participant remained viremic (39 500 copies/mL) due to poor adherence, as confirmed by repeated TDM assessments, including during LEN treatment, which consistently revealed subtherapeutic drug concentrations for all tested agents. The two participants who started with undetectable viremia maintained virological suppression.

### Evaluation of drug resistance in the pol gene

NGS data from plasma and/or PBMCs were obtained for seven participants at T0 and for all at T1 (Figure 1, Table S2). Specifically, at baseline, samples were available for the seven participants with undetectable viremia at T1. For these seven participants, the resistance mutations profile found in PR/RT/IN at T0 was, as expected, complex and comparable to that observed in the historical genotypic resistance test (hGRT) (Table 2, Table S3). No significant changes were generally observed in the mutational patterns both between compartments at the same observation time (T0 or T1) and within the same compartment over time under LEN pressure. The viral blip under LEN pressure (52 copies/mL) was observed at 25 months of LEN treatment in a participant who started LEN in combination with tenofovir disoproxil fumarate/lamivudine, doravirine, fostemsavir and ibalizumab, with an overall GSS of 2.5 (Table 2). Although the detected mutations conferred resistance to part of the regimen, partial activity was preserved through the inclusion of ibalizumab and fostemsavir, allowing maintenance of virological suppression. NGS performed at T1 coincided with the viral blip and showed the additional mutation P225H associated with resistance to doravirine (which was a component of the OBR), detected as a minority variant in PBMCs (intra-patient frequency: 6.2%) (Table 2).

**Table 2.** Resistance profile at baseline (T0) and under LEN pressure (T1) of one individual with a transient blip (A) and one individual with unsuppressed viral load (B) during LEN treatment

A-Individual infected with HIV-1 B subtype with a transient blip (ID-07)								
	HIV-1 RNA (copies/mL)	CD4+ T-cells (cells/mm^3^)	Sample	LEN Major/Accessory	PI Major	NNRTI Major	NRTI Major	INSTI Major/Accessory
hGRT	/	/	Plasma or PBMCs	Not available	V32I, M46I, L76V, V82A, I84V	A98G, K101H, K103H, V108I, E138K, Y181C, G190A	D67N, T69D, K70R, M184V, T215F, T215Y, K219Q	E138T, G140S, Y143C, Q148H/T97A, G149A, G163R
T0	295	469	Plasma			Sample not available		
			PBMCs			Sample not available		
T1 LEN duration (T0-T1) = 25 months	52	721	Plasma	Not interpretable^a^	M46I (98.9%) L76V (98.1%) V82A (98.3%) I84V (97.7%)	A98G (98.7%) K101H (99.0%) K103N (98.2%) V108I (98.7%) Y181C (98.0%) G190A (98.7%)	D67N (98.5%) T69D (97.7%) K70R (98.4%) M184V (98.4%) T215F (97.5%) K219Q (98.4%)	E138K (98.6%) G140S (98.6%) Q148H (97.3%) /T97A (98.4%) G149A (98.5%) G163R (97.5%)
			PBMCs	None/None	V32I (6.2%) M46I (98.5%) L76V (82.2%) V82A (83.5%) I84V (97.1%)	A98G (96.7%) K101H (85.2%) K103H (71.3%) K103N (25.6%) V108I (72.2%) Y181C (88.3%) G190A (88.0%) P225H (6.2%)	D67N (95.0%) T69D (96.1%) K70R (96.2%) M184V (97.1%) T215F (95.7%) K219Q (95.6%)	E138K (60.1%) E138T (5.4%) G140S (66.8%) Q148H (61.3%) /T97A (68.2%) G149A (55.3%) G163R (73.6%)
			Q-distance between plasma versus PBMCs at T1	Not interpretable^a^		PR/RT 0.077		IN 0.098

B-Individual infected with a HIV-1 CRF05_DF with unsuppressed viral load (ID-08)								
	HIV-1 RNA (copies/mL)	CD4+ T-cells (cells/mm^3^)	Sample	LEN Major/Accessory	PI Major	NNRTI Major	NRTI Major	INSTI Major/Accessory
hGRT	/	/	Plasma or PBMCs	Not available	G48S, G48T, I54V	K101E, E138K	T215C, T215Y	E138K/None
T0	23 800	148	Plasma	Sample not available				
			PBMCs	Sample not available				
T1 LEN duration (T0-T1) = 27 months	39 500	58	Plasma	K70H (97.8%) Q67K (98.5%) /None	I54V (98.0%)	K101E (98.6%) E138K (98.9%) Y318F (78.3%)	T215C (94.8%)	None/None
				Fold-Change: 2081 IC50 ± SD (nM): 31.2 ± 2.5				
			PBMCs	K70H (75.6%) Q67K (75.7%) /None	I54V (98.0%)	K101E (96.5%) V106L (16.4%) E138K (97.5%) Y318F (52.9%)	T215C (97.0%)	None/None
			Q-distance between plasma versus PBMCs at T1	CA 0.038		PR/RT 0.021		IN 0.018

A. Drugs administered together with LEN: tenofovir disoproxil fumarate/lamivudine, doravirine, ibalizumab and fostemsavir (GSS: 2.5) for 14 days; tenofovir disoproxil fumarate/emtricitabine, ibalizumab and fostemsavir (GSS: 2.5) for 25 months. B. Drugs administered together with LEN: darunavir/cobicistat, tenofovir alafenamide/emtricitabine and dolutegravir (GSS: 4) for 6 months; tenofovir disoproxil fumarate/lamivudine, doravirine and dolutegravir (GSS: 3.5) for 21 months. ^a^NGS data could not be reliably interpreted owing to low coverage.

The participant with unsuppressed viral load started LEN in combination with darunavir/cobicistat, tenofovir alafenamide/emtricitabine and dolutegravir (GSS: 4), and after 6 months switched to tenofovir disoproxil fumarate/lamivudine, doravirine and dolutegravir (GSS: 3.5) (Table 2). Despite the apparent activity of the administered regimens, viremia remained constantly detectable. At T1, after 27 months of LEN treatment and with a viremia level of 39 500 copies/mL, NGS analysis of the *pol* gene showed additional mutations in RT associated with resistance to doravirine, while no INSTI-resistance mutations were found despite the previous presence of the resistance mutation E138K (Table 2).

### Evaluation of genotypic and phenotypic resistance to LEN

At T0, no major resistance mutations to LEN were detected in any of the seven participants with available NGS data, in either plasma or PBMCs (Table 2). One viremic case harboured the accessory mutation T107A, identified in PBMCs (but not in plasma) as a minority variant (frequency: 5.9%) (Table S3). Phenotypic testing for this participant showed a LEN fold-change value of 0.5, indicating full susceptibility. Baseline phenotypic susceptibility testing confirmed full activity of LEN across the cohort, with a mean fold-change of 1.2 (standard deviation: 0.8; range: 0.5–2.5) (Table 3).

**Table 3.** LEN genotypic and phenotypic resistance profile at baseline

Participants	Subtype	Plasma HIV-1 RNA copies/mL	Fold-Change	IC50 ± SD (nM)	CA major mutation Plasma/PBMC	CA accessory mutation Plasma/PBMC
ID-01	B	2010	1.2	0.018 ± 0.001	None/None	None/None
ID-02	B	25 000	0.5	0.007 ± 0.003	None/None	None/T107A (5.9%)
ID-03	B	642	0.6	0.008 ± 0.003	None/None	None/None
ID-04	B	<50	N.D.	—	−/None	−/None
ID-05	B	884	2.5	0.038 ± 0.017	None/None	None/None
ID-06	B	<50	N.D.	—	−/N.A.	−/N.A.
ID-07	B	295	N.D.	—	—	—
ID-08	CRF05_DF	23 800	N.D.	—	—	—
ID-09	B	121 000	1.3	0.020 ± 0.014	None/None	None/None

NL4-3 (wild-type): Fold-change, 1; IC50 ± SD (nM), 0.015 ± 0.005. N.A., not amplified; N.D., not determined.

In the participant who remained with detectable viremia under LEN pressure, two major LEN resistance mutations—Q67K and K70H—were detected as majority variants in both plasma (frequencies: 98.5% and 97.8%) and PBMCs (75.7% and 75.6%) at T1 (Table 2). Phenotypic testing on plasma sample confirmed a high-level resistance profile, with a LEN fold-change value of 2081.

### Evaluation of intra-host genetic variability

The evolution of intra-host viral diversity over time was investigated in the seven participants for whom a PBMC sample was available at T0 and at T1. In general, the Q-distance found was low (≤0.097), with a median (IQR) of 0.031 (0.019–0.042) for PR/RT, 0.048 (0.022–0.056) for IN and 0.030 (0.019–0.049) for the capsid region, without any significant difference among the analysed regions (*P*-value = 0.911 by Kruskal–Wallis test) (Table S4).

In the participant with persistent viremia for whom both plasma and PBMC samples were available at T1, Q-distance values were evaluated to explore possible compartmentalization of viral quasispecies diversity (Table 2). The Q-distance found was generally low for each region (CA, 0.038; PR/RT, 0.021; IN, 0.018).

## Discussion

The present study assessed genotypic and phenotypic LEN resistance and intra-patient viral evolution over time and across compartments in nine HTE PWH enrolled in the PRESTIGIO Registry who started a regimen containing the capsid inhibitor LEN.

We found an excellent effectiveness of LEN-based regimens in achieving (6/7 PWH) or maintaining (2/2 PWH) virological suppression over a median of about 20 months of treatment in this complex population, in line with that reported in the phase 2/3 CAPELLA trial.^10,19^ A recent study from the same Registry by Clemente *et al*.,^20^ conducted in a larger cohort of 15 viremic and two aviremic individuals (including those described here), reported high rates of virological suppression under LEN treatment, thereby confirming our findings. However, while that study was primarily focused on the durability and viro-immunological outcomes of LEN-containing regimens, our work provides an in-depth virological and molecular characterization, including the longitudinal assessment of resistance dynamics using NGS in paired plasma and PBMC samples, the quantitative analysis of viral quasispecies evolution through Q-distance metrics and *in vitro* phenotypic susceptibility testing.

At baseline, no major LEN RAMs were detected in plasma or PBMCs in any participant. The only exception was the detection of the accessory polymorphic mutation T107A, found as a minority variant (frequency: 5.9%) in the PBMCs of one viremic participant. Our phenotypic analysis revealed that this polymorphism has no impact on LEN susceptibility (fold-change value 0.5), confirming the full susceptibility reported for this variant when present alone (fold-change value 0.6) in the CAPELLA study.^19^ This has been further corroborated by recent data presented by Margot and colleagues at CROI 2026,^21^ demonstrating that T107A/S CA polymorphisms alone do not compromise LEN susceptibility or virological response in clinical practice. The phenotypic profile, together with the maintenance of virological control under LEN treatment in the participant harbouring the T107A mutation, supports its limited clinical impact.

LEN demonstrated notable antiviral activity in this cohort. Two participants who started therapy with undetectable plasma HIV-1 RNA maintained virological suppression throughout follow-up. Among the seven individuals who initiated LEN with unsuppressed viral load, six achieved viral suppression, with only one presenting a transient viral blip (52 copies/mL) during LEN treatment.

In the single participant who remained with sustained viremia despite a LEN-containing regimen, NGS revealed the emergence of Q67K and K70H as majority variants in both plasma and PBMCs after 27 months of therapy, with associated phenotypic resistance showing a >2000-fold reduction in susceptibility. No IN resistance mutations were detected despite the administration of dolutegravir. Although no baseline sequence was available for this participant, the absence of prior LEN exposure and the high prevalence of RAMs at follow-up strongly support their selection under therapeutic pressure. Despite a genotypically active OBR, the development of LEN resistance in this case is likely attributable to suboptimal adherence, which may have created the selective conditions necessary for resistance to emerge and become dominant. Our findings are in line with those found in the CAPELLA study, where the emergence of LEN resistance occurred in participants exposed to prolonged periods of functional LEN monotherapy, often due to inactive OBR or poor adherence.^19^

By evaluating intra-individual PR/RT/IN regions, no relevant differences in resistance profiles were observed both over time and between plasma and PBMC compartments. Additionally, the analysis of intra-host genetic variability through Q-distance measures showed low divergence between T0 and T1 samples, as well as between plasma and cellular compartments in the viremic participant, across the capsid and PR/RT/IN regions. Altogether, the comparable distribution of Q-distances and the lack of significant differences between compartments support the conclusion of a generally homogeneous and stable intra-host viral population during the observation period under LEN pressure. This is compatible with long standing and extensive drug resistance with compensatory changes increasing the stability of the viral quasispecies in HTE PWH.

Our study has several limitations. First, the sample size is small, reflecting the rarity of this population, though the real-world nature of the PRESTIGIO Registry enhances the relevance of these data. Second, not all participants had available samples at both timepoints or from both compartments, limiting the ability to fully reconstruct intra-patient viral evolution. Third, the subtype heterogeneity in our cohort is limited, as eight out of nine PWH were infected with HIV-1 subtype B. Nonetheless, an *in-vitro* study by Naik *et al.* showed that LEN susceptibility and the pattern of selected resistance mutations are similar across different subtypes, thus supporting the broader applicability of our findings.^22^

In conclusion, our findings support the effectiveness of LEN in HTE PWH with MDR, confirming its ability to maintain or restore virological suppression in most cases. The detection of resistance mutations, such as Q67K and K70H, in the single case of persistent viremia under LEN treatment supports concerns regarding the genetic barrier of LEN. Nevertheless, suboptimal adherence leading to functional LEN monotherapy represents the most plausible driver of resistance, as observed in the CAPELLA study, although the contribution of undetected pre-existing mutations cannot be fully ruled out. These findings highlight the need for close virological monitoring together with optimized companion drugs, in case of low adherent HTE PWH with MDR with unsuppressed viremia.

## GenBank accession numbers

The data that support the findings of this study are available from the corresponding author upon reasonable request. All sequences obtained from this study have been deposited into GenBank (accession numbers: PZ024067-PZ024088, PZ670555).

## Supplementary Material

dkag335_Supplementary_Data

## Supplementary data

Tables S1 to S4 is available as Supplementary data at *JAC* Online.
