# Supplementary material for: Virological outcomes, resistance profile and viral evolution of lenacapavir-based regimens in heavily treatment-experienced people: data from the PRESTIGIO Registry
Source: J Antimicrob Chemother. 2026 Oct 28;81(11):dkag335. doi: 10.1093/jac/dkag335 (PMC13714547; doi:10.1093/jac/dkag335)
Supplement: Supplementary file 1 [file dkag335_supplementary_data.docx]

**Supplementary Tables:**

| **Table S1. Drugs administered together with LEN at the moment of switch.** | | | | | | | | | | |
| --- | --- | --- | --- | --- | --- | --- | --- | --- | --- | --- |
| **Participants** | **EI** | **FI** | **NRTI** | **NNRTI** | **INSTI** | **PI** | **mAB** | **GSS^a^** | **Number of drugs with LEN** | **Number of backbone changes^b^** |
| ID-01 |  | ENF  (0) | 3TC/TDF (0/0.5) | DOR  (0.5) |  |  |  | 1 | 4 | 1 |
| ID-02 | MVC  (0) |  | FTC/TAF (0/0) | DOR  (0) |  | DRV/COBI (0) | IBA  (1) | 1 | 6 | 1 |
| ID-03 |  |  | FTC/TAF (0/0) | DOR  (0) |  | DRV/COBI (0) |  | 0 | 4 | 0 |
| ID-04 |  | ENF  (1) | FTC/TAF (0/0.5) | DOR  (0.5) |  | DRV/COBI (0) |  | 2 | 5 | 1 |
| ID-05 |  |  | FTC/TAF (0/0) |  |  | DRV/COBI (1) |  | 1 | 3 | 4 |
| ID-06 |  |  | FTC/TAF (0/0) | DOR  (0.5) |  | DRV/COBI (1) |  | 1.5 | 4 | 0 |
| ID-07 | FTR  (1) |  | 3TC/TDF (0/0.5) | DOR  (0) |  |  | IBA  (1) | 2.5 | 5 | 1 |
| ID-08 |  |  | FTC/TAF (1/1) |  | DTG  (1) | DRV/COBI (1) |  | 4 | 4 | 2 |
| ID-09 | FTR  (1) |  | FTC/TAF (0/0.5) |  | DTG  (0) | DRV/RTV (0.5) | IBA  (1) | 3 | 6 | 0 |
| Values in parentheses indicate the individual Genotypic Susceptibility Score (GSS) for each drug.  ^a^GSS of the first regimen administered together with lenacapavir. ^b^Number of backbone regimen changes administered together with lenacapavir over the period between the first (T0) and second (T1) sample collected for the study. 3TC, lamivudine; DOR, doravirine; DRV/COBI, darunavir/cobicistat; DRV/RTV, darunavir/ritonavir; DTG, dolutegravir; EI, entry inhibitors; ENF, enfuvirtide; FI, fusion inhibitors; FTC, emtricitabine; FTR, fostemsavir; IBA, ibalizumab; INSTI, integrase strand transfer inhibitor; LEN, lenacapavir; mAB, monoclonal antibody; MVC, maraviroc; NNRTI, non-nucleoside reverse transcriptase inhibitor; NRTI, nucleoside reverse transcriptase inhibitor; PI, protease inhibitor; TAF, tenofovir alafenamide; TDF, tenofovir disoproxil fumarate. | | | | | | | | | | |

| **Table S2. Overview of samples availability and virological (genotypic and phenotypic) analyses performed for each participant at baseline (T0) and follow-up (T1) in HTE individuals from the PRESTIGIO Registry who started a lenacapavir-based regimen.** | | | | | | |  |
| --- | --- | --- | --- | --- | --- | --- | --- |
|  |  |  |  |  |  |  |  |
| **Participants** | **Baseline (T0)** | | | **Follow-up (T1)** | | |  |
|  | **PBMCs** | **PLASMA** | | **PBMCs** | **PLASMA** | |  |
|  | **PR/RT/IN & CA Genotyping** | **PR/RT/IN & CA**  **Genotyping** | **Phenotyping**  ***(In vitro Lenacapavir assay)*** | **PR/RT/IN & CA**  **Genotyping** | **PR/RT/IN & CA**  **Genotyping** | **Phenotyping**  ***(In vitro Lenacapavir assay)*** |  |
|  |  |  |  |  |  |  |  |
| ID-01 | ✓ | ✓ | ✓ | ✓ | N.Av. | N.Ap. (VL < 50 copies/mL) |  |
| ID-02 | ✓ | ✓^b^ | ✓ | ✓ | N.Av. | N.Ap. (VL < 50 copies/mL) |  |
| ID-03 | ✓ | ✓ | ✓ | ✓ | N.Av. | N.Ap. (VL < 50 copies/mL) |  |
| ID-04 | ✓ | N.Av. | N.Ap. (VL < 50 copies/mL) | ✓ | N.Av. | N.Ap. (VL < 50 copies/mL) |  |
| ID-05 | ✓ | ✓ | ✓ | ✓ | N.Av. | N.Ap. (VL < 50 copies/mL) |  |
| ID-06 | ✓^a^ | N.Av. | N.Ap. (VL < 50 copies/mL) | ✓ | N.Av. | N.Ap. (VL < 50 copies/mL) |  |
| ID-07 | N.Av. | N.Av. | N.Av. | ✓ | ✓^a^ | N.Ap. (VL = 52 copies/mL) |  |
| ID-08 | N.Av. | N.Av. | N.Av. | ✓ | ✓ | ✓ |  |
| ID-09 | ✓ | ✓^c^ | ✓ | ✓ | N.Av. | N.Ap. (VL < 50 copies/mL) |  |
| ^a^Successful genotyping for PR/RT/IN and not for CA. ^b^Successful genotyping for IN and CA and not for PR/RT. ^c^Successful genotyping only for CA and not for PR/RT/IN. CA, capsid; HTE, highly treatment‐experienced; IN, integrase; N.Ap., not applicable; N.Av., not available; PR/RT, protease/reverse transcriptase; VL, viral load; PBMCs, peripheral blood mononuclear cells. Checkmark (✓) indicates successfully performed genotyping or phenotypic assay. | | | | | | |  |

| **Table S3. Genotypic resistance profile of the seven participants who achieved (n=5) or maintained virological suppression (n=2) under lenacapavir treatment.** | | | | | | | | | | | | |
| --- | --- | --- | --- | --- | --- | --- | --- | --- | --- | --- | --- | --- |
| Participant | Time | **HIV-1 RNA (copies/mL)** | **CD4+ T-cells (cells/mm^3^)** | | **Sample** | **LEN Major/Accessory** | **PI Major** | **NNRTI Major** | | | **NRTI Major** | **INSTI Major/Accessory** |
| **ID-01**  **Subtype B** | **hGRT** | / | / | | **Plasma or PBMCs** | Not available | V32I, L33F, M46I, M46L, I54A, T74P, I84V, L90M | K101E, E138A,V179F, Y181V, G190A | | | M41L, D67N, K70Q, K70T, V75M, F77L, M184I, L210W, T215C, T215F, T215Y, K219R | E138A, G140S, Y143C, Y143H, Y143R, Q148H /T97A |
|  | **T0** | 2,010 | 111 | | **Plasma** | None/None | V32I (72.0%), M46L (98.6%), I54A (98.4%), I84V (99.0%), L90M (98.6%) | K103N (97.2%), E138A (98.7%), G190A (99.1%) | | | M41L (98.6%), E44D (98.9%), D67N (94.5%), K70Q (98.5%), V75M (98.8%), F77L (98.9%), M184I (98.8%), L210W (58.7%), T215Y (95.8%) | E138A (98.5%), G140S (98.5%), Q148H (97.1%)/T97A (97.9%), G149A (98.5%) |
|  |  |  |  |  | **PBMCs** | None/None | V32I (28.1%), M46L (12.5%), I54A (39.6%), I54V (58.5%), L76V (10.8%), I84V (97.3%), L90M (97.6%) | E138A (97.7%), G190A (49.4%) | | | M41L (95.1%), E44D (83.3%), D67N (95.2%), K70Q (14.6%), V75M (97.5%), F77L (97.5%), M184I (97.7%), L210W (70.2%), T215Y (94.5%), K219R (85.9%) | E138A(66.5%), G140S(62.1%), Q148H(60.1%)/T97A(67.6%), G149A(60.7%) |
|  | **T1** | TND | 147 | | **PBMCs** | None/None | V32I (6.5%), M46L (7.5%), I54A (98.3%), I84V (96.8%), L90M (98.0%) | E138A (97.8%), Y188L (9.1%), G190A (10.0%) | | | M41L (97.3%), E44D (97.2%), D67N (94.7%), K70Q (14.1%), V75M (97.7%), F77L (97.9%), M184I (98.2%), L210W (82.0%), T215Y (97.2%), K219R (86.1%) | E138A (94.4%), G140S (94.3%), Q148H (93.1%)/  T97A (93.4%), G149A (94.1%) |
|  |  |  |  |  |  |  |  |  |  |  |  |  |
| **ID-02**  **Subtype B** | **hGRT** | / | / | | **Plasma or PBMCs** | Not available | V32I, L33F, K43T, M46I, M46L, I54A, I54L, I54T, I54V, T74P, V82F, I84V, L90M | A98G, E138A, Y181C, Y181I, Y181V, G190A, M230L | | | M41L, D67G, K70Q, L74V, V75I, V75M, F77L, M184V, L210W, T215F, T215Y, K219E | G140S, Q148H/T97A |
|  | **T0** | 25,000 | 41 | | **Plasma** | None/None | Not Amplified | Not Amplified | | | Not Amplified | G140S (98.5%), Q148H (97.7%)/T97A (97.7%) |
|  |  |  |  |  | **PBMCs** | None/T107A (5.9%) | None | V108I (34.5%), Y181C (5.7%), G190A (5.5%) | | | M41L (42.9%), L74V (5.5%), L210W (40.1%), T215Y (30.0%) | G140S (48.7%), Q148H (51.4%)/T97A (33.5%) |
|  | **T1** | TND | 1159 | | **PBMCs** | None/None | None | K103N (31.8%) | | | M41L (11.1%), M184V (9.5%), L210W (11.7%), T215Y (7.9%) | G140S (30.2%), Q148H (28.6%)/None |
|  |  |  |  |  |  |  |  |  |  |  |  |  |
| **ID-03**  **Subtype B** | **hGRT** | / | / | | **Plasma or PBMCs** | Not available | L10F, V32I, L33F, K43T, I54L, T74P, I84V | A98G, K103N, Y181C, P225H | | | M41L, D67G, K70E, K70R, K70T, L74I, M184V, T215F, T215Y, K219E, K219N | E138A, G140S, Q148H/H51Y, |
|  | **T0** | 642 | 173 | | **Plasma** | None/None | V32I (98.3%), I54L (97.8%), I84V (97.3%) | A98G (98.4%), K103N (98.0%), Y181C (97.4%) | | | M41L (69.2%), D67G (94.8%), S68G (97.0%), K70R (41.3%), L74I (83.2%), M184V (98.0%), T215Y (94.5%), K219E (98.1%) | G140S (96.9%), Q148H (93.0%)/H51Y (93.2%), T97A (98.3%) |
|  |  |  |  |  | **PBMCs** | None/None | V32I (14.1%), I54L (14.5%), I84V (15.5%) | A98G (62.1%), K103N (16.8%), Y181C (17.1%) | | | M41L (48.1%), D67G (64.1%), S68G (61.3%), K70A (24.9%), K70R (11.8%), K70T (20.5%), L74I (20.0%), M184V (64.6%), T215F (15.6%), T215V (22.6%), T215Y (15.0%), K219E (14.8%) | G140S (85.2%), Q148H (83.8%)/H51Y (80.2%), T97A (83.4%) |
|  | **T1** | TND | 287 | | **PBMCs** | None/None | V32I (8.2%), I84V (6.7%) | A98G (61.5%), Y181C (9.3%) | | | M41L (27.7%), D67G (49.6%), S68G (48.0%), K70A (26.2%), K70T (27.5%), L74I (9.7%), M184V (60.2%), T215F (25.6%), T215L (8.0%), T215S (5.7%), T215Y (7.1%) | E138A (5.1%), G140S (46.5%), Q148H (45.9%)/H51Y (36.6%), T97A (40.9%) |
|  |  |  |  |  |  |  |  |  |  |  |  |  |
| **ID-04**  **Subtype B** | **hGRT** | / | / | | **Plasma or PBMCs** | Not available | V32I, L33F, I47V, I50L, I54L, I54M, Q58E, I84V, L90M | K101E, Y181I | | | M41L, M184V, T215F | N155H/None |
|  | **T0** | <50 | 518 | | **PBMCs** | None/None | V32I (5.6%), L90M (93.6%) | V108I (7.9%) | | | M41L (5.7%) | N155H (12.3%)/None |
|  | **T1** | <50 | 630 | | **PBMCs** | None/None | V32I (11.0%), I47V (8.2%), I54M (8.7%), I84V (10.5%), L90M (95.2%) | K101E (18.4%), V108I (7.6%), Y181I (19.4%) | | | M41L (25.2%), M184V (16.2%), T215F (13.8%) | G140R (7.2%), N155H (13.1%)/None |
|  |  |  |  |  |  |  |  |  |  |  |  |  |
| **ID-05**  **Subtype B** | **hGRT** | / | / | | **Plasma or PBMCs** | Not available | G48M, F53L, I54A, V82A | L100I, K103N | | | M41L, D67G, T69D, V75M, M184V, L210W, T215D, T215Y, K219R | None/E157Q |
|  | **T0** | 884 | 187 | | **Plasma** | None/None | None | None | | | M41L (96.8%), D67G (95.7%), T69D (97.8%), V75M (97.8%), M184V (97.8%), L210W (98.0%), T215D (96.2%), K219R (97.8%) | None/E157Q (98.3%) |
|  |  |  |  |  | **PBMCs** | None/None | G48M (24.7%), I54A (21.8%), V82A (29.1%) | L100I (25.3%), K103N (24.2%) | | | M41L (62.9%), E44D (23.9%), D67G (67.4%), T69D (58.6%), K70R (35.4%), V75M (73.8%), M184V (65.2%), L210W (66.0%), T215D (41.2%), T215Y (19.1%), K219R (64.7%) | N155H (29.6%)/E157Q (98.4%) |
|  | **T1** | <50 | 276 | | **PBMCs** | None/None | G48M (18.1%), I54A (16.9%), V82A (25.7%) | None | | | M41L (73.6%), D67G (82.0%), T69D (76.7%), K70R (9.2%), V75M (53.3%), F77L (10.6%), M184V (76.9%), L210W (77.1%), T215D (46.8%), T215Y (23.8%), K219R (75.8%) | None/E157Q (98.3%) |
|  |  |  |  |  |  |  |  |  |  |  |  |  |
| **ID-06**  **Subtype B** | **hGRT** | / | / | | **Plasma or PBMCs** | Not available | M46I, G73A, L90M | L100I, V108I | | | M41L, D67S, L74V, M184V, L210W, T215Y | E138K, Q148R /None |
|  | **T0** | <50 | 566 | | **PBMCs** | Not Amplified | M46I (29.4%), I54V (7.5%), V82A (10.6%), L90M (32.8%) | L100I (6.4%), K103N (15.9%), V108I (5.7%), E138A (30.1%), Y181C (8.9%) | | | M41L (71.4%), E44D (63.4%), D67N (34.6%), T69D (21.7%), K70R (5.9%), L74V (10.7%), M184V (23.5%), L210W (55.6%), T215Y (64.0%) | None/None |
|  | **T1** | <50 | 507 | | **PBMCs** | None/None | M46I (36.4%), V82A (6.7%), L90M (38.5%) | L100I(7.0%), K103N(21.8%), V106A(7.3%), V108I(9.4%), E138A(28.1%), Y181C(8.8%) | | | M41L (64.9%), E44D (65.7%), D67N (36.5%), T69D (21.1%), K70R (5.1%), L74V (11.0%), M184V (27.7%), L210W (60.1%), T215Y (58.1%) | None/None |
|  |  |  |  |  |  |  |  |  |  |  |  |  |
| **ID-09**  **Subtype B** | **hGRT** | / | / | | **Plasma or PBMCs** | Not available | L10F, L33F, G73S, I84V, L90M | K103N, Y181C | | | M41L, L74V, M184V, T215C, T215Y | E138K, G140S, Q148H/T97A, |
|  | **T0** | 121000 | 275 | | **Plasma** | None/None | Not available | | | | | |
|  |  |  |  |  | **PBMCs** | None/None | I84V (97.0%) | K103N (97.0%), Y181I (95.3%) | | | M41L (96.9%), D67E (5.8%), D67N (89.8%), T215Y (89.7%) | E138K (97.5%), G140S (97.7%), Q148H (95.8%)/T97A (96.4%), A128T (21.0%) |
|  | **T1** | TND | 209 | | **PBMCs** | None/None | I84V (93.3%) | K103N (93.5%), Y181I (89.9%) | | | M41L (97.0%), D67E (5.3%), D67N (82.8%), T215Y (88.9%) | E138K (89.0%), G140S (95.1%), Q148H (93.5%)/T97A (92.2%), A128T (10.9%) |
| In the table are reported all resistance mutations detected in historical genotypic resistance test (hGRT) and by NGS using a cut-off >5%. For the following participants resistance profile through NGS was determined only in PBMCs because their viremia levels were below 50 copies/mL: ID-01 (T1), ID-02 (T1), ID-03 (T1), ID-04 (T0, T1), ID-05 (T1), ID-06 (T0, T1), ID-09 (T1). TND, target not detected. | | | | | | | | | | | | |

| **Table S4. Intra-host viral diversity over time.** | | | |
| --- | --- | --- | --- |
| **ID participant** | **Q-distance in PBMCs between T0 and T1** | | |
|  | CA | PR/RT | IN |
| ID-01 | 0.071 | 0.097 | 0.048 |
| ID-02 | 0.055 | 0.043 | 0.053 |
| ID-03 | 0.032 | 0.031 | 0.058 |
| ID-04 | 0.012 | 0.026 | 0.024 |
| ID-05 | 0.027 | 0.041 | 0.058 |
| ID-06 | Not Available | 0.012 | 0.007 |
| ID-09 | 0.017 | 0.011 | 0.019 |
| Overall (median [IQR]) | 0.030 (0.019-0.049) | 0.031 (0.019-0.042) | 0.048 (0.022-0.056) |
| Intra-host genetic variability under lenacapavir pressure was evaluated in PBMCs by using Q-distance in the seven participants with undetectable viremia. The table reports the Q-distances found in PR/RT, IN and the capsid regions per each participant and overall (expressed as median [IQR]). Differences in the median Q-distances were evaluated overall (Kruskal-Wallis test) and as pairs (Dunn’s post-hoc test): CA vs. PR/RT vs. IN p-value = 0.911; CA vs. PR/RT p-value = 0.404; CA vs. IN p-value = 0.432; PR/RT vs. IN p-value = 0.334. After Bonferroni correction all the p values were 1.000. | | | |
